# How Face Masks Affect the Use of Echolocation by Individuals With Visual Impairments During COVID-19: International Cross-sectional Online Survey

**DOI:** 10.2196/39366

**Published:** 2022-10-25

**Authors:** Chantal Kreidy, Natalina Martiniello, Joseph Paul Nemargut, Walter Wittich

**Affiliations:** 1 School of Optometry University of Montreal Montréal, QC Canada; 2 Centre for Interdisciplinary Research in Rehabilitation of Greater Montreal Montreal, QC Canada; 3 Centre de Réadaptation Lethbridge-Layton-Mackay du Centres Intégrés Universitaires de Santé et de Services Sociaux du Centre-Ouest-de-l’Île-de-Montréal Montreal, QC Canada; 4 Institut Nazareth et Louis-Braille du Centres Intégrés de Santé et de Services Sociaux de la Montérégie-Centre Longueuil, QC Canada

**Keywords:** visual impairment, echolocation, COVID-19, orientation and mobility, vision rehabilitation, online survey, rehabilitation, face mask, visual disability, vision disorder, quality of life, health intervention

## Abstract

**Background:**

Although a critical safety measure, preliminary studies have suggested that the use of a face mask may pose a problem for some users with disabilities. To date, little is known about how the wearing of a traditional face mask may pose a barrier to individuals with visual impairments who draw on auditory cues and echolocation techniques during independent travel.

**Objective:**

The goal of this study was to document the difficulties, if any, encountered during orientation and mobility due to the use of a face mask during the COVID-19 pandemic and the strategies used to address these barriers.

**Methods:**

In total, 135 individuals aged 18 years and older who self-identified as being blind, being deafblind, or having low vision and who could communicate in either English or French completed an anonymous cross-sectional online survey between March 29 and August 23, 2021.

**Results:**

In total, 135 respondents (n=52, 38.5%, men; n=83, 61.5%, women) between the ages of 18 and 79 (mean 48.22, SD 14.48) years participated. Overall, 78 (57.7%) self-identified as blind and 57 (42.3%) as having low vision. In addition, 13 (9.6%) identified as having a combined vision and hearing loss and 3 (2.2%) as deafblind. The most common face coverings used were cloth (n=119, 88.1%) and surgical masks (n=74, 54.8%). Among the barriers raised, participants highlighted that face masks made it more difficult to locate people (n=86, 63.7%), communicate with others (n=101, 74.8%), and locate landmarks (n=82, 60.7%). Although the percentage of those who used a white cane before the pandemic did not substantially change, 6 (14.6%) of the 41 participants who were guide dog users prior to the pandemic reported no longer working with a guide dog at the time of the survey. Moreover, although guide dog users reported the highest level of confidence with independent travel before the pandemic, they indicated the lowest level of confidence a year after the pandemic began.

**Conclusions:**

These results suggest that participants were less able to draw on nonvisual cues during independent travel and social interactions due to the use of a facemask, contributing to a reduction in perceived self-confidence and independence. Findings inform the development of evidence-based recommendations to address identified barriers.

## Introduction

### Background

The COVID-19 pandemic has had a significant impact on the lives of individuals worldwide. Measures have been implemented to mitigate the spread of the virus, including physical distancing, the use of face masks, and avoiding all nonessential travel [1]. Though critical, such measures also adversely impact the quality of life and increase social isolation [2]. It is projected that the pandemic will carry negative consequences for the mental and physical health and self-care of individuals, especially for persons with disabilities, including those with visual impairments [3-5]. Many individuals with visual impairments (ie, those who are blind or who have low vision) already experience higher levels of social isolation due to factors such as inaccessible physical environments and the inability to rely on vision during independent travel [6-11]. Vision rehabilitation practitioners are health care professionals who provide training and support for individuals with a congenital or acquired visual impairment. A variety of techniques and strategies are used to regain or maintain independence for activities of daily living, including the use of a white cane for independent travel in indoor and outdoor environments. Although an essential safety measure, initial evidence indicates that the use of a face mask may impair the ability of individuals with visual impairments to use echolocation and other auditory strategies by either muffling self-generated sounds (eg, speech) or obstructing vital environmental cues that reach the ears (eg, the sound of parallel traffic) [12,13]. Despite the urgency of these concerns, there is a lack of research on the effect of face masks on echolocation or on the potential safety implications that may arise during the COVID-19 pandemic. These questions will remain important to facilitate planning for additional waves of the current pandemic and for strengthening strategies to better prepare for future pandemics. This study explored the nature and extent of problems caused by face masks within the visually impaired population and gathered information about the strategies that individuals with visual impairments have used to circumvent these barriers. The findings will be used to develop recommendations for rehabilitation centers and governments on how to effectively assist clients during both current and future pandemics.

### The Population: Individuals With Visual Impairments

According to the World Health Organization (WHO), it is estimated that there are 405.5 million individuals with mild-to-severe visual impairments worldwide and 36 million who are blind [14]. The term “visual impairment” includes both low vision and blindness. As the majority of the cortex is devoted to visual perception and processing, vision is the primary sense that is used for performing most daily tasks [11]. Individuals with visual impairment may therefore encounter a variety of obstacles in their daily lives. Vision loss is associated with decreased mobility, thus contributing to higher levels of social isolation and depressive symptoms for those who have not adopted compensatory techniques [6-11,15]. To mitigate these challenges, individuals with visual impairments use a variety of techniques, including the use of a white cane or a guide dog [16].

### Echolocation and Auditory Cues as Compensatory Strategies

One of the major challenges faced by individuals with visual impairments relates to orientation and mobility. For individuals with little or no vision, the ability to perceive objects from a distance and to navigate within space depends primarily on auditory and spatial information [17]. It has now been established that the human brain has a remarkable ability to adapt to changes in the environment due to its plasticity. For example, individuals with congenital blindness experience cortical reorganization early in life, which allows them to recruit the unused visual cortex during nonvisual tasks [18-21]. Studies have shown that such cortical plasticity is associated with enhanced auditory spatial abilities and echolocation skills and that this improved performance is experience dependent [14].

In general, echolocation represents the ability for an individual to locate objects and obstacles by interpreting the reflected echoes of sounds that bounce off them. There are two overarching categories of echolocation. On the one hand, active echolocation refers to the self-generation of sounds via tongue clicking, whistling, humming, and talking or externally via finger snapping, the tapping of a white cane, or other noises to gain information on object/obstacle localization through reflected echoes. On the other hand, passive echolocation refers to when individuals use the reflected echoes of sounds that naturally occur and that provide auditory cues and landmarks to help interpret the environment (eg, the sound of parallel traffic, people talking, or sounds that emerge from nearby businesses) [19,20,22,23]. Evidence suggests that passive echolocation is more commonly used than active echolocation because individuals with visual impairments are often reluctant to self-generate sounds due to the stigma associated with these behaviors [24]. Data show that echolocation allows visually impaired individuals to locate objects, differentiate between objects of various sizes and shapes, and in some cases differentiate between objects of various textures [20]. Auditory cues (eg, the sound of others speaking) also provide vital information to help orient individuals to others during social interactions. Although echolocation is generally unconsciously used by individuals with visual impairments, active echolocation techniques must typically be taught [25,26]. For the purposes of this study, echolocation is defined as both the active and the passive use of sounds for travel and social interactions. In addition to the use of sound during independent travel, individuals with visual impairments also draw on other sources of sensory information, including texture, olfactory, and tactile cues and residual vision [12,16,27].

### Face Masks and COVID-19

COVID-19 is transmitted through respiratory droplets or through the mouth, nose, or eyes after direct contact with a contaminated surface [1,3]. Recent studies indicate that individuals with a visual impairment are more susceptible to COVID-19 due, in part, to the fact that they may rely on assistance of others for certain tasks and may be less able to easily avoid others in public in order to maintain physical distancing [4,5,26]. Initial reports suggest that face masks introduce new barriers for individuals with a visual impairment who use echolocation during independent travel to detect obstacles and to navigate more easily [14]. From a social interaction perspective, it is unknown whether the use of face masks may impair the ability of blind individuals to use auditory cues to determine where others are located, which may further increase anxiety when navigating in public during the pandemic and increase social isolation. Though not focusing on face masks, prior studies have demonstrated that difficulties due to audition (eg, loud environments) can be cognitively demanding and stressful to visually impaired individuals (and even more so for those with dual sensory impairments) who may not be aware of when they are being spoken to or who may be less able to rely on visual cues during social interactions [28]. Though anecdotal, it has also been suggested that the use of face masks may pose unique communication challenges for guide dog users by muffling the vocalization of handlers when commands are being communicated [29,30]. Although guide dogs are not trained to rely on facial expressions and instead rely on a combination of verbal and physical commands [31,32], it is known that dogs read subtle muscle changes that indicate a twitch or a smile, which may facilitate communication [29,33]. The inability to use echolocation is especially concerning, as individuals with visual impairments are less able to rely on alternative options, such as the use of sighted guides and volunteers during the pandemic, due to physical distancing measures [3,5,26,34,35]. There is thus an urgent need for research that explores the potential impact of face masks on both independent travel and social interaction in order to guide rehabilitation practitioners and others on how to best advice and support individuals with visual impairments during this time.

### Objectives and Research Questions

Although a critical safety measure, recent studies have explored how the use of traditionally designed face masks may pose unique challenges to other disability groups. Most notably, face masks prevent individuals with hearing impairments from relying on lip- and speech-reading techniques, leading to the development of clear face masks as more effective alternatives [36-38]. Although concerns have been raised about the use of face masks among individuals with visual impairments [12,13], there is no prior research that gathers evidence on the nature and extent of this issue or the possible solutions that exist. The objectives of this study were therefore to:

- Explore the nature and extent of problems caused by face masks among individuals with a visual impairment.
- Determine whether demographic variables (eg, level of vision, age of onset, visual diagnosis, mobility aids used, and age) are characteristics of participants who experience these problems.
- Understand the strategies that individuals have used to overcome these barriers.

## Methods

### Ethical Considerations

Ethical approval was obtained from the Institutional Review Board of the Université de Montréal in March 2021 (CERC-21-019-D).

### Participants

#### Eligibility Criteria

To take part in this study, participants had to be at least 18 years of age; self-identify as an individual who is blind, is deafblind, or has low vision; and be able to communicate in English or French (one of the survey language options). Given that the use of face masks may pose barriers to individuals regardless of where they reside and that data were gathered through an online survey, no restriction based on geographic location was imposed.

#### Sample Size

Given the lack of prior research in this domain, this study was exploratory in nature. Therefore, no traditional power analysis was conducted during the proposal stage. Based on a previous international online survey geared toward individuals who are blind, are deafblind, or have low vision, conducted by members of this research team [39], a sample size of more than 150 was estimated. Though obtaining a sufficient sample size is often difficult in blindness research due to the low incidence of visual impairment [40], members of the research team have drawn on an extensive list of social media platforms to assist with recruitment, which has resulted in sample sizes of more than 400 in similar research based on a survey instrument [39]. Additionally, experts with visual impairments served as core members of the research team to guide research design, ensure greater accessibility and inclusion, and facilitate recruitment.

#### Recruitment

Recruitment was carried out using 3 main approaches: social media announcements and outreach to blindness consumer groups, collaboration through vision rehabilitation centers, and snowball sampling [41]. Participants were primarily invited through announcements on social media platforms geared toward individuals who are blind, have low vision, or are deafblind (Facebook, Twitter, and email groups). A list of over 150 social media groups was compiled by the research team to assist with recruitment. Where moderator approval was required before posting to a specific social media group, this permission was requested in advance.

In addition, the study was promoted through different media outreach activities, including interviews on blindness media platforms [42,43]. Finally, snowball sampling (whereby participants were asked to refer anyone else they know who may be interested) provided additional reach beyond these initial contacts [44].

### Materials and Procedure

Data were gathered through an anonymous, online, voluntary survey that required an average of 36 minutes (SD 29.75) to complete. It contained 35-58 questions in total, depending on participant responses, because certain questions were conditional on the participant’s response. Although the options to allow participants to go back or perform a completeness check before submitting their answers were not available, the survey was built in a way that participants could not go to the next question without completing the previous one. It was first piloted by 2 users (1 blind and 1 with low vision) to ensure accessibility. The survey was available between March 29 and August 23, 2021. Participants provided informed consent in accordance with the Declaration of Helsinki and Public Health by selecting the “I agree” button to proceed with the survey [45].

We did not report unique visitors’ information. The survey was anonymized, and as such we did not track IP addresses, nor were cookies or other mechanisms used to block repeated submissions from a single IP address, computer, or web browser. There was a risk that either mechanism might block legitimate users from accessing the survey (eg, multiple respondents from a single rehabilitation center). Moreover, we do not have information about the unique survey visitors versus unique site visitors because the survey was advertised through a wide range of platforms (and via email links); therefore, they did not all arrive at the survey from a single site that could be tracked directly. However, our participation rate for the survey (visitors who agreed to participate/unique first survey page visitors) was 9%. Our completion rate for the survey (users who finished the survey/who agreed to participate) was 49.1%.

#### Survey Instrument

The survey was developed and executed using the LimeSurvey software package and hosted on servers provided by the Université de Montréal. This platform was chosen because it is known to be accessible to users who are blind or have low vision and enables the collection of data through a secured, encrypted internet channel. The survey was available in both English and French, and responses were collected through a combination of closed- and open-ended questions. At the start of the survey, participants were asked to answer questions about their age and level of vision to ensure that they met the eligibility criteria, prior to moving on to subsequent sections.

Section 1 of the survey (14-20 questions) gathered demographic information from participants, including information about the level and nature of visual impairment and living arrangements both before and since the pandemic began. Section 2 (9-15 questions) gathered information about the orientation and mobility history and behaviors of participants, including mobility aids used (eg, guide dog, white cane), their level of confidence with independent travel both before and after the pandemic began, and echolocation techniques used by participants while traveling. Section 3 (7-13 questions) gathered information about the barriers encountered due to the use of a face mask, including barriers related to independent travel (the ability to use auditory and other nonvisual cues in the environment) and those related to social interaction (the ability to communicate with others and maintain physical distancing). Section 4 (2-7 questions) asked participants what strategies they have used to address these barriers, if any. The final section provided participants with the option to indicate whether they wished to receive a summary of the results, participate in a draw, or provide any final comments about their experiences while using a face mask. Participants had the option at the end of the survey to include their name in a draw for the chance to win a CA $100 (US $72.71) Amazon gift card and to request an email summary of the results once the study is complete, but in such cases, contact information was separated from survey responses (see Multimedia Appendix 1 for the full survey instrument).

#### Data Analysis

The aim of this study was to explore the barriers encountered due to the use of a face mask among individuals with visual impairments and the strategies used to address identified barriers. For these reasons, descriptive statistical analysis for quantitative data was primarily used. Mixed within-between analyses of variance were performed to test the existence of a statistically significant difference before and after the onset of the pandemic, as well as between participant groups based on demographic variables. Only completed surveys were analyzed.

## Results

### Demographics

**Figure 1:**
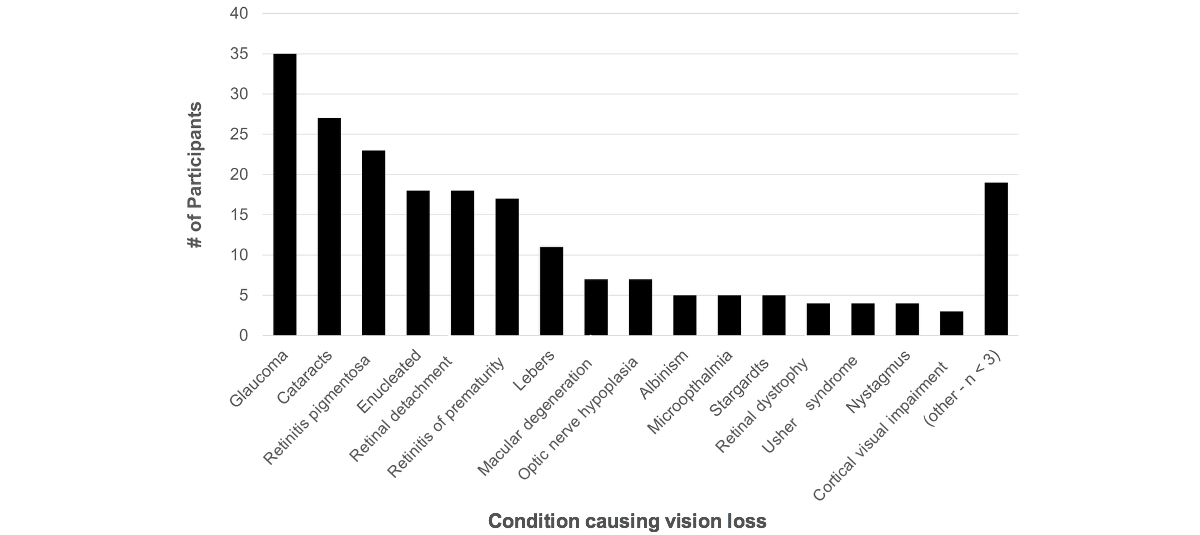
Number of participants reporting each of the most common medical diagnoses. N>135 as many participants reported having multiple diagnoses.

Table 1 summarizes the demographic characteristics of the 135 participants in the study, who were between the ages of 18 and 79 (mean 48.22, SD 14.47) years. Age of onset for visual impairment ranged from birth to 59 years of age (mean 9.45, SD 14.24). Figure 1 shows the number of participants who reported the most common diagnoses. Of the 16 (11.9%) participants who reported a hearing impairment, age of onset of hearing impairment ranged from birth to 66 years of age (mean 31.04, SD 25.89).

**Table 1:** Demographic characteristics of participants (N=135).

Characteristics		Participants, n (%)
**Sex**		
	Female	83 (61.5)
	Male	52 (38.5)
**Gender**		
	Female	86 (63.7)
	Male	48 (35.6)
	Nonbinary	1 (0.7)
**Level of vision impairment^a^**		
	Blind	78 (57.8)
	Low vision	57 (42.2)
**Level of hearing impairment^a^**		
	Hard of hearing	13 (9.6)
	Deaf	3 (2.2)
**Country of residence**		
	Canada	85 (63.0)
	United States	32 (23.7)
	France	11 (8.1)
	England	2 (1.5)
	Morocco	1 (0.7)
	Italy	1 (0.7)
	Australia	1 (0.7)
	Sri Lanka	1 (0.7)
	Tunisia	1 (0.7)
**Highest completed level of education**		
	Some high school	6 (4.4)
	High school diploma	14 (10.4)
	Vocational/professional education	6 (4.4)
	Community college or Collège d'enseignement général et professionnel diploma	31 (23.0)
	Undergraduate degree (eg, bachelor’s)	42 (31.1)
	Graduate degree (eg, master’s or PhD)	36 (26.7)
**Employment status**		
	Self-employed	10 (7.4)
	Full-time employee	42 (31.1)
	Part-time employee	13 (9.6)
	Student	15 (11.1)
	Retired	29 (21.5)
	Unemployed	26 (19.3)
**Face coverings used^b^**		
	Cloth masks	119 (88.1)
	Surgical masks	74 (54.8)
	N95 respirators	21 (15.6)
	Face shields	11 (8.1)
	Glasses	6 (4.4)

^a^Some participants may have reported both vision and hearing losses; these 2 categories are not mutually exclusive.

^b^Participants could mark all that apply for face coverings used.

### Orientation and Mobility

**Figure 2:**
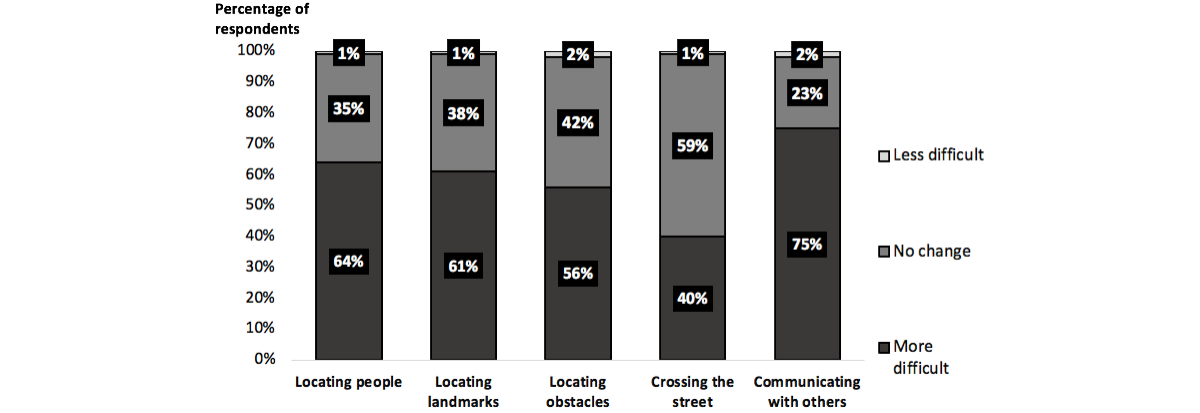
Selected orientation and mobility tasks and the change in difficulty experienced by travelers on account of the use of a face mask.

Table 2 summarizes the travel techniques used by the participants in the study. Findings indicate that 85 (63%) participants used a white cane pre–COVID-19 compared to 84 (62.21%) participants who reported still using a white cane post–COVID-19. Conversely, 41 (30.4%) participants identified as guide dog users pre–COVID-19 compared with only 35 (25.9%) who reported working with a guide dog after the pandemic began. The majority of participants found that common orientation and mobility tasks, such as locating people and landmarks, were made more difficult by the use of face masks (see Figure 2).

**Table 2:** Travel techniques and tools used for orientation and mobility, and navigation (N=135).

Technique		Participants, n (%)^a^
**Passive echolocation**		
	In outdoor environments	109 (80.7)
	In indoor environments	100 (74.1)
**Active echolocation**		
	Cane tapping	66 (48.9)
	Finger snapping	18 (13.3)
	Tongue clicking	17 (12.6)
	Tactile feedback (from white cane or feet)	105 (77.8)
	Olfactory cues	91 (67.4)
	Estimation of distance	84 (62.2)
	Residual vision	66 (48.9)

^a^The 135 participants may have selected more than 1 travel technique.

### Impact on Travel Frequency and Confidence

**Figure 3:**
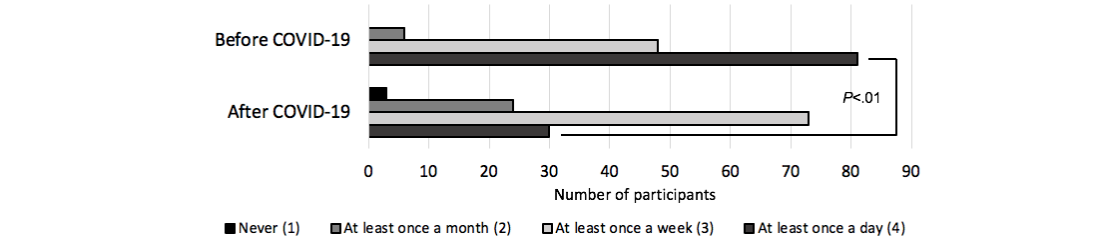
Frequency of travel prior to versus during the COVID-19 pandemic.

The frequency at which participants traveled independently significantly decreased between the period before and after the pandemic began (see Figure 3). A repeated-measures analysis of covariance revealed that there was a statistically significant difference in the frequency of travel before versus after the pandemic began (*F*_1,128_=6.51, *P*=.012, η^2^=0.014) but not when controlling for age (*F*_1,128_=0.192, *P*=.66).

**Figure 4:**
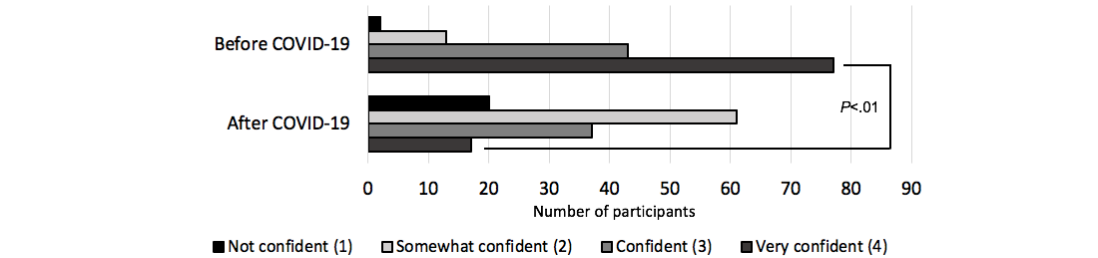
Degree of confidence in independent travel prior to versus during the COVID-19 pandemic.

The confidence with which participants traveled independently (measured on a scale wherein 1=not confident at all, 2=somewhat confident, 3=confident, and 4=very confident) also decreased between the period before and after the pandemic began (see Figure 4). A repeated-measures analysis revealed that there was a statistically significant difference in the frequency of travel before versus after the pandemic (*F*_1,133_=24.09, *P*<.001, η^2^=0.051) but not when controlling for age (*F*_1,133_=0.930, *P*=.34). The most common explanations given by those who indicated that they were “not confident’ or were only “somewhat confident” included difficulty communicating with other people (n=86, 63.7%); barriers to using sighted guide assistance, when required, due to physical distancing requirements (n=58, 43%); impairment of the ability to detect landmarks, such as bus shelters or intersecting hallways (n=57, 42.2%); impairment of the ability to hear ambient noises in the environment (n=55, 40.7%); and interference with the use of active echolocation techniques, such as tongue clicking (n=28, 20.7%).

**Figure 5:**
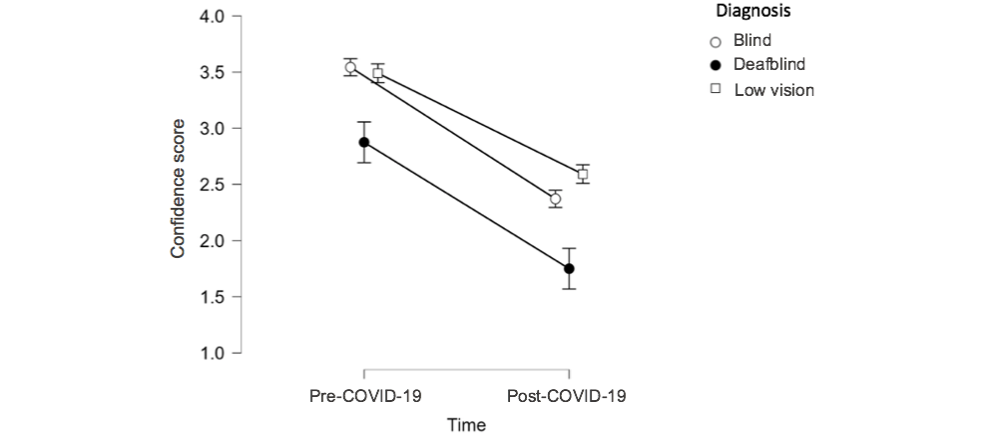
Level of confidence pre- and post–COVID-19 by level of vision loss. Confidence was measured on a scale wherein 1=not confident at all, 2=somewhat confident, 3=confident, and 4=very confident.

The level of confidence of participants who were deafblind (compared to those who were blind or who had low vision) was particularly negatively impacted (see Figure 5). A repeated-measures analysis of variance showed that mean confidence differed significantly between time points for all groups (*F*_1,132_=133.02, *P*<.001, η^2^=0.229), with those who self-identified as deafblind experiencing a significantly greater loss of confidence in comparison with participants who had low vision and were blind.

**Figure 6:**
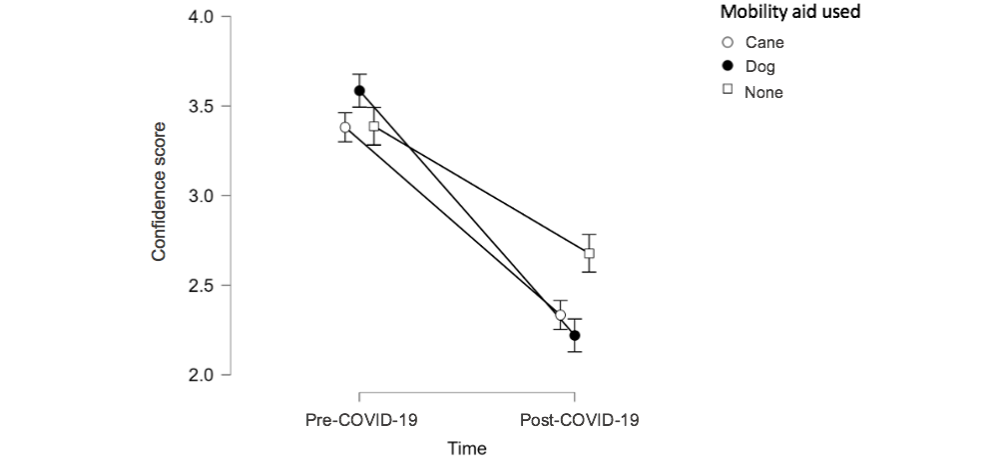
Level of confidence pre- and post–COVID-19 by mobility aid. Confidence was measured on a scale wherein 1=not confident at all, 2=somewhat confident, 3=confident, and 4=very confident.

Likewise, the level of confidence of guide dog users was especially negatively impacted compared to the level of confidence of those using a cane or no mobility aids (Figure 6). A repeated-measures analysis of variance showed that mean confidence differed significantly between time points for all groups (*F*_1,132_=179.77, *P*<.001, η^2^=0.275), with guide dog users exhibiting a significantly greater loss of confidence in comparison with cane users and those who did not use a mobility aid.

### Strategies for Overcoming Barriers

Table 3 outlines the different strategies that participants have used to overcome the barriers posed by the use of face masks. The results show that most of the strategies used were the use of other senses, delivery services, and sighted guides (where a person with a visual impairment holds the arm of a sighted guide while walking through a physical environment). In addition, few people asked for help from rehabilitation centers (n=7, 5.2%). However, a recurring theme in the comments from participants was that it was more difficult to get sighted guide assistance since the beginning of the pandemic, as expressed by these quotes:

While traveling independently has been made more difficult, getting assistances has also diminished. People are hesitant to touch or be close.

Before the pandemic people were more agreeable to providing human guide assistance.

**Table 3:** Number of participants identifying various barriers to travel while wearing a face mask, and the strategies used to overcome the identified barriers (N=135)^a^.

Barriers	Other senses, n (%)	Delivery services, n (%)	Sighted guide, n (%)	Phone app, n (%)	Rehabilitation services, n (%)	Nothing, n (%)
Communicate with others (n=17)	7 (41.2)	5 (29.4)	5 (29.4)	3 (17.6)	0	5 (29.4)
Locate people around (n=87)	40 (46.0)	31 (35.6)	38 (43.7)	8 (9.2)	1 (1.1)	26 (29.9)
Locate landmarks (n=82)	43 (52.4)	38 (46.3)	48 (58.5)	15 (18.3)	7 (8.5)	16 (19.5)
Locate obstacles (n=76)	44 (57.9)	30 (39.5)	42 (55.3)	12 (15.8)	4 (5.3)	16 (21.1)
Crossing streets (n=55)	27 (49.1)	26 (47.3)	32 (58.2)	9 (16.4)	6 (10.9)	12 (21.8)

^a^The counts and percentages in each row add up to more than the number of respondents who identified a particular barrier, as respondents used more than 1 strategy to overcome said barrier.

## Discussion

### Principal Findings

The aim of this study was to explore the challenges caused by the use of traditional face masks for individuals with visual impairments and to determine the strategies used to overcome these barriers. Overall, findings confirm that individuals with visual impairments continue to experience a variety of barriers related to orientation and mobility during the pandemic and that this is especially true for those with lower levels of vision and those who use a guide dog. The results also highlight a significant decrease in confidence and in the frequency of travel that has persisted since the pandemic began, raising concerns about the ability of individuals with visual impairments to access services and maintain social participation as the pandemic persists. Although participants highlight the use of sighted guide as a strategy to overcome barriers during independent travel, access to sighted support is limited due to physical distancing measures. Moreover, although the majority of participants rely on online and delivery services in place of independent travel, the accessibility of these online services remains a persistent barrier despite the existence of international web accessibility standards. Despite these challenges, most participants have not sought support from existing rehabilitation services.

### Problems Caused by Face Masks

Although no significant decrease in white-cane usage was reported after the pandemic began, 6 of the 41 guide dog users reported no longer using a guide dog now. In some instances, this decrease may be due to the natural retirement of service dogs due to advancing age or other circumstances not directly related to the pandemic, such as behavioral or health issues [46]. However, evidence also indicates that border closures during the pandemic have significantly impeded the ability of guide dog handlers to receive support when issues arise with guide dog usage and have prevented those who require successor dogs from pursuing training [47,48]. To address these persisting barriers, several dog guide–training organizations have implemented virtual services to provide training support from a distance, where feasible, or are in the process of hiring staff who live closer to geographic areas with a high density of guide dog users [49]. Despite these measures, guide dog organizations report a notable decrease in the number of service teams they have been able to serve during the pandemic and mounting wait lists that will require several years to resolve [46,49]. Moreover, border closures have led to an increase in requests for domestic guide dog training and support. The Canadian National Institute for the Blind Foundation, which serves individuals within Canada, for instance, recently noted that demand for services has increased by at least 300% during the pandemic [34]. Although there is no clear guideline to address such service disruptions, advocates continue to highlight the need for governments to recognize dog guide training as an essential service during current and future pandemics.

The inability to access support explains, in part, the significant decrease in confidence experienced by guide dog users. These issues are compounded by the inability of guide dog handlers to regularly work their dogs during lockdowns and other isolation measures, as routine reinforcement in training is essential to maintain guide work skills, particularly for dogs that are younger and more recently attributed [50,51]. These findings highlight the need for orientation and mobility specialists and guide dog–training programs to proactively collaborate to ensure that clients who use guide dogs can access emergency support during times of a pandemic and international crises. In particular, evidence of best practices for virtual service delivery and methods for coordinating with local rehabilitation centers, where feasible, will be important to address. To this end, a number of dog guide organizations have developed online tools to educate members of the public on how to assist guide dog users during the pandemic while maintaining physical distancing [52]. In addition to this vital information, there is a need for an increased number of guide dog service providers as well [46,53,54].

Participants indicated that the wearing of a traditional face mask impeded the ability to locate others in the environment, locate landmarks, and communicate with others. Although the wearing of a face mask appears to interfere with the use of echolocation, participants also highlight that the ability to draw on other senses, including olfactory cues from the environment, is diminished. The results also demonstrate that there has been a significant decrease in the frequency and level of confidence during independent travel before and after the beginning of the pandemic. These findings highlight the need to reinforce and improve universal accessibility measures within physical environments, particularly as individuals may be limited even further in their ability to draw on nonvisual senses and may need to rely even more heavily on environmental cues, such as tactile floor indicators, braille and large-print signage, and audible pedestrian signals [55]. Although many countries, including Canada [56] and the United States [57], maintain legislation that requires that public places incorporate universal design principles, it is evident that such accessibility measures are not consistently integrated into all spaces. The findings of this research are particularly noteworthy in Canada, where the newly implemented Accessible Canada Act aims to address existing gaps within accessibility and inclusion at the federal level [58].

### Impact of Demographic Variables

Results show that although guide dog users were the most confident while traveling alone prior to the pandemic, they were the least confident after the pandemic began. Prospective guide dog users must typically demonstrate existing orientation and mobility skills in order to qualify for a guide dog, which may explain why this group exhibited the highest level of confidence prior to the pandemic when compared to other mobility aid users [59]. This is due, in part, to the fact that dog guides are trained to navigate around obstacles, but it is the responsibility of the handler to maintain a mental map of where they wish to go and to communicate those instructions to their dog [60]. However, physical distancing measures introduced with the pandemic may be especially challenging for this population, given that dog guides are not typically trained to maintain physical distancing while in public places [61]. This further highlights the need for those around guide dog users to maintain physical distancing and to recognize these challenges when providing assistance.

In addition, Figure 5 indicates to which extent participants with lower levels of vision, including those with dual sensory impairments, reported experiencing the most significant decrease in the level of confidence since the pandemic began. This finding might be due to the fact that these participants have less vision to rely on, which increases challenges if the use of echolocation and other auditory cues is limited. This is especially noteworthy, given that there is a growing prevalence of individuals with age-related dual sensory impairment due to a rapidly aging population in developed countries [62]. Governments, including rehabilitation agencies and allied services, should ensure that there are virtual opportunities to maintain social participation and access essential services, especially since many individuals with dual sensory loss are also in older age groups that already experience higher levels of isolation [63]. In response, some rehabilitation centers have implemented virtual peer support groups and social activities where individuals can socialize and receive support [64-67]. Although such measures are no doubt crucial during the pandemic, the enhancement of telehealth and rehabilitation services provide new opportunities to more effectively address the needs of individuals who live outside urban areas, including indigenous communities that often remain underserved and isolated. It will be important to consider the long-term maintenance of such new measures even after the pandemic subsides.

### Strategies That Individuals Have Used to Overcome the Barriers Raised

The most common strategies used to circumvent identified barriers included the use of a sighted guide, the use of other senses, and the use of online delivery services. However, participants reported the limited availability of sighted assistance during the pandemic due to physical distancing measures. Moreover, many online delivery services remain either partially or wholly inaccessible to users with visual impairments who access content through electronic braille, auditory, or magnification software [68,69]. Screen-reading software is one of the most common assistive technologies used by individuals with visual impairments to access information [69]. Although international standards for web accessibility exist [70], the accessibility of websites and apps remains a pervasive problem, limiting usability for individuals with diverse needs. For example, many platforms incorporate images and other graphical content that cannot be interpreted by screen-reading software, without the inclusion of alternative captioned text to make this content accessible to users without sight. When such inaccessible features are embedded within online delivery services (eg, online restaurant menus), such services are inherently unusable to individuals with visual impairments [71,72]. Ensuring that such universal design standards are proactively incorporated and maintained across all online services is especially vital during times of international crises and pandemics, when individuals may have even less access to services to address their basic safety needs. Indeed, the International Council on English Braille recently called upon governments and organizations to ensure the accessibility of information, pointing not only to access to services but also to information about rising infection rates and safety measures [73]. The findings of this study also indicate that only a minority of participants (5%) turned to vision rehabilitation services for assistance, perhaps suggesting that individuals are unaware of how such agencies can provide support. Although not a replacement for mainstream accessibility and inclusion measures, for example, some organizations provide services to assist individuals with daily tasks, ranging from grocery shopping to reading mail. This study further underscores the need for vision rehabilitation centers to go beyond the services they can provide and to develop public education resources to train mainstream service providers (health care providers, store clerks, etc) on how to effectively guide and assist individuals with visual impairments while maintaining physical distancing measures. As evidenced by some efforts in this domain, such training could incorporate strategies for providing effective verbal information and the use of modified sighted guide techniques [52,74,75].

### Limitations

Although this is among the first studies to explore the impact of traditional face masks on orientation and mobility during COVID-19 among individuals with visual impairments, a number of limitations should be noted. First, language could have had an impact on the number of participants as the survey and the media announcements were only available in French or English. Second, data collection primarily consisted of announcements circulated through online social media platforms and email mailing lists. It is therefore possible that this may have limited participation from those within the blind and low-vision community who do not have access to technology or who lack the technical competence to complete an online survey. However, to circumvent these concerns, participants had the option to request assistance by phone, and efforts were made to advertise the survey to diverse groups within the population, including consumer groups geared toward older adults with acquired vision loss, and through more traditional methods, such as radio and TV announcements. In addition, given the descriptive nature of the study, we did not calculate a power analysis in order to determine an optimal sample size. Instead, we followed the recruitment approach of a previous study [39], where we obtained an impressive sample of 466 participants with visual impairments. Although we did not recruit as many participants as in the previous study, we were content with a sample of 135 participants.

### Implications for Practice

These results contribute to the development of recommendations on how to address identified barriers. These recommendations include the accessibility of online and remote services (a health equity issue) by reviewing websites to ensure they comply with provincial and federal accessibility legislation and international web accessibility standards [55,70,76]. This includes the need to ensure that accessible information about the pandemic (including infection rates and safety measures) is available to those within the community who are most vulnerable, including people with disabilities [73]. Training for sighted guide and service providers on human guide techniques that respect physical distancing measures must be expanded [77]. Research and practice initiatives are needed that more closely explore the feasibility of where and when to provide remote assistance for rehabilitation and orientation and mobility services [64,67]. Finally, it is critical to reconsider designing guide dog–training services to better support and address user needs during times of international crises, including the feasibility of remote assistance, strategies for maintaining physical distancing when using a guide dog, and methods for educating the public on how to interact with guide dog users during COVID-19.

### Conclusion

The pandemic has introduced new problems for all individuals but poses even greater issues for equity-seeking groups who already encounter barriers within society. For persons with visual impairments (including those who are deafblind), navigating through physical environments may be impeded by various accessibility challenges (eg, inaccessible environments). During the pandemic, the use of a traditional face mask may pose new problems, including the inability to rely on echolocation techniques and external auditory cues from the environment. For those with little or no sight, and for those who use guide dogs, such challenges may pose significant safety concerns and lower the level of confidence during independent travel. Despite the barriers raised, no existing research has explored the impact of traditional face masks on mobility among individuals with visual impairments during the COVID-19 pandemic. This study highlights the extent of the difficulties that individuals with vision impairment have continued to face during the pandemic and stresses the importance of implementing solutions to better circumvent identified barriers. Although the wearing of face masks is essential to prevent the transmission of COVID-19, it is evident from these findings that a number of strategies can address the difficulties encountered by users with visual impairments. Ensuring the accessibility of online services, expanding access to remote and virtual support, and educating members of the public will address many of the barriers raised to improve the lived experiences of individuals both during and after the current pandemic.

## Appendix

Multimedia Appendix 1 Survey instrument.

## Data Availability

The anonymized data set as well as its corresponding data labels and questionnaire items are available through the Open Science Framework.
