# Supplementary material for: How Face Masks Affect the Use of Echolocation by Individuals With Visual Impairments During COVID-19: International Cross-sectional Online Survey
Source: Interact J Med Res. 2022 Oct 25;11(2):e39366. doi: 10.2196/39366 (PMC9604170; doi:10.2196/39366)
Supplement: Multimedia Appendix 1 [file ijmr_v11i2e39366_app1.docx]

**Multimedia Appendix 1**

**35 to 58 questions (depending on the participant’s responses)**

[Information regarding requirements to set on survey fields is in square brackets]

[Note: Where there are ‘conditional’ questions that depend on earlier answers, the ‘conditional’ questions have to be placed on a separate page. Having questions appear/disappear on a single page will be inaccessible for screen reader users]

# Question to confirm eligibility (this question will appear before the consent form)

To participate in this survey

- You must be at least 18 years or older
- You must have a visual impairment (low vision, blindness or deafblindness)
- You must have been using a face mask or face covering at least some of the time during the pandemic

Do you meet these eligibility requirements?

[radio button, choose one]

( ) Yes

( ) No

If “no” they will be taken to a thank you page.

If “yes” they will be taken to the consent form. If they agree to the terms and conditions listed in the consent form they will be then taken to the survey. I they do not agree they will be sent to a thank you page.

# Demographics

This section includes 14 to 20 questions. These questions ask about your background (for example, age, visual impairment and living arrangements)

What is your current age? (open-ended; in years) (Q1)

[Dropdown list]

Which of these terms do you most identify with? (radio button, choose one.) (Q2)

( ) I have low vision

( ) I am blind

Do you also have a hearing impairment? (radio button, choose one.) (Q3)

( ) Yes, I have a hearing impairment

( ) Yes, I am deaf

( ) I do not have a hearing impairment

If participant selects “low vision + hearing impairment” or “deafblind” in the vision impairment question, then ask:

What is the cause of your hearing impairment, if known? (open-ended) [Text field, no max length, not required] (Q4)

If Hearing Impairment = any of the YES options, then ask: At what age were you diagnosed with your hearing impairment, if known? (Enter 0 if you were diagnosed at birth.) (open-ended) [Text field, not required, max length 3, accept numbers only] (Q5)

Biological sex at birth: (Q6)

( ) male

( ) female

( ) intersex

( ) prefer not to say

[Radio button group, required]

Gender identity: (Q7)

listbox of options, taken from: https://www.surveymonkey.com/curiosity/ask-survey-questions-sexual-orientation-gender-identity/

Visual diagnoses (if known) (select all that apply): (Q8)

[Group of check boxes, required, may select multiple]

( ) Albinism

( ) Cataracts

( ) Diabetic retinopathy

( ) eye(s) removed

( ) Glaucoma

( ) Macular degeneration

( ) Optic nerve hypoplasia

( ) Retinal detachment

( ) Retinitis pigmentosa

( ) Retinopathy of prematurity

( ) Stargart’s disease

( ) Usher’s syndrome

( ) Other (please describe)

( ) I don’t know

At what age were you diagnosed with your visual condition? (if known) (enter 0 if diagnosed at birth) (Q9)

[Text field, not required, max length 3, accept numbers only]

How would you describe your current visual impairment (in your better eye)? (choose one.)

[Radio button group, required] (Q10)

( ) I have a central visual impairment

( ) I have a peripheral (side vision) impairment

( ) I have a general visual impairment (both peripheral and central)

Please list any other diagnoses that you feel may effect your orientation and mobility: [Multiple line text area, not required] (Q11)

What is your highest level of completed education? (Q12)

[Radio button group; required]

( ) Some high school

( ) High school diploma

( ) Vocational education (e.g. apprenticeship or trade education)

( ) Community college diploma or CEGEP diploma

( ) Undergraduate degree (e.g. Bachelor’s degree)

( ) Postgraduate degree (e.g. Master’s degree or PhD)

Current employment: (Q13)

[Radio button group; required]

( ) Self-employed

( ) Full-time employed

( ) Part-time employed

( ) Student

( ) Retired

( ) Unemployed

In what city were you living before the pandemic began? (Q14)

[Text field, required]

In what country were you living before the pandemic began? (Q15)

[Text field, required]

Have you moved since the pandemic began? (Q16)

[Radio button group, required]

( ) Yes

( ) No

Question asked only if participant selected that they moved sometime after the pandemic began:

You indicated that you moved after the start of the pandemic. In what city are you currently living? (Q17)

[Text box, required]

You indicated that you moved after the start of the pandemic. In what country are you currently living? (Q18)

[Text box, required]

What were your reasons for moving after the pandemic began? (Select all that apply.) (Q19)

[Check box group: required]

( ) To live closer to family and friends

( ) Changed economic or financial circumstances

( ) Changed health or disability status

( ) Loss of employment

( ) Disruption to educational status or change of educational format (e.g. distance education)

( ) Other (please describe.) ___________________

How long have you been living in your current city?: (Q20)

[Radio button group, required]

( ) Six months or less

( ) Between six months and a year

( ) More than a year

# Orientation and Mobility

This section includes 9 to 15 questions about your Orientation and Mobility situation.

What type of face mask or face covering have you been using at least some of the time since the pandemic began? (Q21)

( ) Cloth face mask or face covering

( ) Surgical face mask

( ) N95/KN95

( ) Face shield

( ) Eye goggles/protection

( ) Other

What modes of transportation did you use before the pandemic? (Q22)

[Group of check boxes, multiple select allowed, required]

( ) Public transportation (e.g. bus, subway, train)

( ) taxi/ride share

( ) Specialized accessible transportation

( ) Walk

( ) Cycle

( ) Someone I know drives me (e.g. friend, family, colleague, volunteer)

( ) Not applicable

( ) Other

What modes of transportation have you used since the pandemic began? (Q23)

[Group of check boxes, multiple select allowed, required]

( ) Public transportation (e.g. bus, subway, train)

( ) Uber/taxi/ride share

( ) Specialized accessible transportation

( ) Walk

( ) Cycle

( ) Someone I know drives me (e.g. friend, family, colleague, volunteer)

( ) Not applicable

( ) Other

Rate your level of confidence when traveling independently before the pandemic began:

[Radio button group, required] (Q24)

( ) Very confident

( ) Confident

( ) Somewhat confident

( ) Not confident at all

Rate your level of confidence when traveling independently since the pandemic began

: (Q25)

( ) Very confident

( ) Confident

( ) Somewhat confident

( ) Not confident at all

If participant chose any of the not confident options in above question (not confident, somewhat confident) then ask:

all You stated that you do not feel confident when traveling since the pandemic began. What factors have contributed to your lack of confidence? (select that apply.) (Q26)

( ) The use of a face mask impairs my ability to hear ambient noises in the environment while traveling (e.g. the sound of parallel traffic, footsteps, etc.)

( ) The use of a face mask reduces my ability to detect certain landmarks using my hearing (bus shelters, intersecting hallways, etc.)

( ) The use of a face mask prevents me from using self-generated echolocation techniques (e.g. finger snapping, tongue clicking)

( ) The use of a face mask makes it harder for me to communicate with my guide dog

( ) The use of a face mask makes it harder for me to communicate with a sighted guide

( ) The use of a face mask makes it harder for me to hear what people are saying while trying to talk to them

( ) The use of a face mask makes it harder for me to avoid people in public places (maintaining social distance)

( ) Face masks do not effect my abilities

( ) Other (related to wearing a face mask) (please describe)

How often did you travel outside your home before the pandemic began? (Q27)

[Radio button group, required]

( ) At least once per day

( ) Once a week

( ) Several times per week

( ) Several times a month

( ) Never

( ) Other (please describe)

Since the pandemic began, how often have you been traveling outside your home? (Q28)

[Radio button group, required]

( ) At least once per day

( ) Once a week

( ) Several times per week

( ) Several times a month

( ) Never

( ) Other (please describe)

By what means did you typically navigate, before the pandemic? Please check all that apply: (29)

[Check box group, multiple selection permitted, not required]

( ) Using smartphone apps

( ) Using specialized GPS devices for the blind

( ) White cane (for mobility)

( ) White cane (for identification only)

( ) Guide dog

( ) Sighted guide / asking friends or family to travel with you

( ) Independently travel by memorizing routes

( ) Mostly travel independently, but sometimes ask for help from others to make sure that I am on the right path

( ) Other: (please describe)

Since the pandemic began, by what means have you typically been navigating? Please check all that apply: (Q30)

[Check box group, multiple selection permitted, not required]

( ) Using smartphone apps

( ) Using specialized GPS devices for the blind

( ) White cane (for mobility)

( ) White cane (for identification only)

( ) Guide dog

( ) Sighted guide / asking friends or family to travel with you

( ) Independently travel by memorizing routes

( ) Mostly travel independently, but sometimes ask for help from others to make sure that I am on the right path

( ) Other: (please describe)

If “Means of Mobility” in previous question included “Guide Dog”: How long have you had a guide dog? (Q31)

[Radio button group, required]

( ) Less than 1 year

( ) 1-2 years

( ) 3-6 years

( ) More than 6 years

If “Means of Mobility” included “Guide Dog”: Are you currently working with your first guide dog? (Q32)

[Radio button group, required]

( ) Yes

( ) No

If “Means of Mobility” included “White Cane (Mobility)”: How long have you been using a white cane? (Q33)

[Radio button group, required]

( ) Less than 1 year

( ) 1-2 years

( ) 3-6 years

( ) More than 6 years

If “Means of Mobility” included “White Cane (Mobility)”: Did you receive training on the use of the white cane from a blindness professional? (Q34)

[Radio button group, required]

( ) Yes

( ) No, I did not want training.

( ) No, I wanted training but did not know where to get it.

( ) Other: ____

If “First Guide Dog” = No” then: How long have you and your current guide dog been a team?

[Radio button group, required] (Q35)

( ) Less than 1 year

( ) 1-2 years

( ) 3-6 years

( ) More than 6 years

# Impact of Wearing a Face Mask or face covering (7 to 13)

Below is a list of techniques that you might use to aid in your orientation and mobility. These techniques might include what is called “echolocation”: the use of sound to locate objects or obstacles in the environment.

Please select all that you use (at least some of the time) when travelling. (Q36)

[Group of check boxes, multiple select permitted]

( ) Echolocation through tongue clicking

( ) Echolocation through finger snapping

( ) Echolocation through the sound of cane tapping

( ) Drawing on olfactory cues (smell of a bakery, fries, coffee, etc)

( ) Estimating the distance travelled

( ) Drawing on auditory cues from external sound sources outdoors (e.g. sound of parallel traffic, sound of footsteps, sound of bus shelter)

( ) Drawing on auditory cues from external sound sources indoors (footsteps, ventilation, flushing toilets, etc.)

( ) Tactile feedback from your cane or your feet

( ) Residual vision

( ) Other: (please describe) _____

If “Travel Techniques” includes any of the self-generated options (options 1-3) in above question, then ask:

Since the pandemic began, have you found that a face mask or face covering impairs your ability to use echolocation techniques (self-generated sounds such as tongue clicking, finger snapping, cane tapping) while traveling? (Q37)

[Radio button group, required]

( ) Yes

( ) No

If yes to above question then ask:

Please describe how the use of a face mask or face covering has effected your ability to use self-generated echolocation techniques (e.g. finger snapping, tongue-clicking, sound of cane tapping): (open-ended field) (Q38)

If “travel techniques” includes olfactory, then ask:

Since the pandemic began, have you found that a face mask or face covering impairs your ability to rely on olfactory cues (e.g. the scent of a bakery) when traveling?

[Radio button group, required] (Q39)

( ) Yes

( ) No

If “Travel Techniques” includes use of external auditory sounds in q*, then ask:

Since the pandemic began, have you found that a face mask or face covering impairs your ability to use echolocation techniques (auditory cues from the external environment such as the sound of parallel traffic) while traveling? (Q40)

[Radio button group, required]

( ) Yes

( ) No

Please describe how the use of a face mask or face covering has effected your ability to rely on external auditory cues (e.g. sound of parallel traffic): (open-ended) (Q41)

If “Guide Dog” = “Yes”: Do you find that a face mask or face covering impairs your ability to communicate verbally with your guide dog while you are travelling? (Q42)

[Radio button group, required]

( ) Yes

( ) No

If yes to above, then ask:

How does wearing a face mask or face covering effect your ability to communicate with your guide dog? (open-ended field) (Q43)

Have you found that the use of face mask or face covering makes it more or less difficult for you to determine the location of people you may be interacting with in public? (Q44)

[Radio button]

( ) A face mask makes it more difficult to locate people I interact with in public

( ) A face mask makes it somewhat more difficult to locate people I interact with in public

( ) A face mask makes no difference when locating people I interact with in public

( ) A face mask makes it somewhat less difficult to locate people I interact with in public

( ) A face mask makes it less difficult to locate people I interact with in public

Have you found that the use of face mask or face covering makes it more or less difficult for you to avoid obstacles when walking? (Q45)

[Radio button]

( ) A face mask makes it more difficult to avoid obstacles when walking

( ) A face mask makes it somewhat more difficult to avoid obstacles when walking

( ) A face mask makes no difference when avoiding obstacles when walking

( ) A face mask makes it somewhat less difficult to avoid obstacles when walking

( ) A face mask makes it less difficult to avoid obstacles when walking

Have you found that the use of face mask or face covering makes it more or less difficult for you to use auditory cues to cross streets when walking? (Q46)

[Radio button]

( ) A face mask makes it more difficult to use auditory cues for crossing streets when walking

( ) A face mask makes it somewhat more difficult to use auditory cues for crossing streets when walking

( ) A face mask makes no difference when using auditory cues for crossing streets when walking

( ) A face mask makes it somewhat less difficult to use auditory cues for crossing streets when walking

( ) A face mask makes it less difficult to use auditory cues for crossing streets when walking

Have you found that the use of a face mask or face covering makes it more or less difficult for you to hear what others are saying when speaking to you? (Q47)

[Radio button]

( ) A face mask makes it more difficult to hear what others are saying when speaking to me

( ) A face mask makes it somewhat more difficult to hear what others are saying when speaking to me

( ) A face mask makes no difference when hearing what others are saying when speaking to me

( ) A face mask makes it somewhat less difficult to hear what others are saying when speaking to me

( ) A face mask makes it less difficult to hear what others are saying when speaking to me

Have you found that the use of a face mask or face covering makes it more or less difficult to determine the location of landmarks and cues while walking? (Q48)

[Radio button]

( ) A face mask makes it more difficult to determine the location of landmarks and cues while walking

( ) A face mask makes it somewhat more difficult to determine the location of landmarks and cues while walking

( ) A face mask makes no difference when determining the location of landmarks and cues while walking

( ) A face mask makes it somewhat less difficult to determine the location of landmarks and cues while walking

( ) A face mask makes it less difficult to determine the location of landmarks and cues while walking

# Strategies Used

You’re almost done! This section includes 2 to 7 questions that ask about the strategies you’ve used since the pandemic started.

If “Guide Dog Communication Difficulty” == “yes” then: You said that you have some difficulty communicating verbally with your guide dog when wearing a face mask. Which of the following strategies have you used to work around that problem? (Q49)

[Check box group, required]

( ) Using in-person sighted assistance

( ) Using a remote sighted assistance “app” on my smartphone (e.g. AIRA, Be My Eyes)

( ) Using my other senses

( ) Staying home, and relying more on delivery services

( ) Contacting a rehabilitation center for training and support

( ) None – I have simply accepted it

( ) Other: __________________________

If “Location of People” == “more difficult” then: You said that you have more difficulty determining where people are located when wearing a face mask. Which of the following strategies have you used to work around that problem? (Q50)

[Check box group, required]

( ) Using in-person sighted assistance

( ) Using a remote sighted assistance “app” on my smartphone (e.g. AIRA, Be My Eyes)

( ) Using my other senses

( ) Staying home, and relying more on delivery services

( ) Contacting a rehabilitation center for training and support

( ) None – I have simply accepted it

( ) Other: __________________________

If “Avoiding Obstacles” == “more difficult” then: You said that you have more difficulty avoiding obstacles when wearing a face mask. Which of the following strategies have you used to work around that problem? (Q51)

[Check box group, required]

( ) Using in-person sighted assistance

( ) Using a remote sighted assistance “app” on my smartphone (e.g. AIRA, Be My Eyes)

( ) Using my other senses

( ) Staying home, and relying more on delivery services

( ) Contacting a rehabilitation center for training and support

( ) None – I have simply accepted it

( ) Other: __________________________

If “Determining location of landmarks” == “more difficult” then: You said that you have more difficulty determining the location of landmarks and cues when wearing a face mask. Which of the following strategies have you used to work around that problem? (Q52)

[Check box group, required]

( ) Using in-person sighted assistance

( ) Using a remote sighted assistance “app” on my smartphone (e.g. AIRA, Be My Eyes)

( ) Using my other senses

( ) Staying home, and relying more on delivery services

( ) Contacting a rehabilitation center for training and support

( ) None – I have simply accepted it

( ) Other: __________________________

If “Auditory Cues when Crossing” == “more difficult” then: You said that you have more difficulty using auditory cues when crossing streets when wearing a face mask. Which of the following strategies have you used to work around that problem? (Q53)

[Check box group, required]

( ) Using in-person sighted assistance

( ) Using a remote sighted assistance “app” on my smartphone (e.g. AIRA, Be My Eyes)

( ) Using my other senses

( ) Staying home, and relying more on delivery services

( ) Contacting a rehabilitation center for training and support

( ) None – I have simply accepted it

( ) Other: __________________________

Are there any other challenges you have experienced, relating to your orientation and mobility, when wearing a face mask? If so, please describe them and what, if anything, you have been able to do to overcome them. (Q54)

[Open text area]

Please describe whether the type of face mask or face covering you use (e.g: cloth, surgical, etc.)

Do you feel that the material of the face mask or face covering that you use (for example, cloth vs. surgical) impacts your orientation and mobility (for example, makes it harder to hear sounds in the environment)? If so, please describe. (Q55)

[Long text - Optional]

# Conclusion

This is the final section! This section includes 3 questions to gather your final thoughts.

Is there anything else you would like to share about your experience traveling since the pandemic began? (please describe.) (Q56)

[Open text area]

As a token of our appreciation for your participation, would you like to be entered into a draw to receive a $100 Amazon gift card? (Q57)

( ) Yes

( ) No

Would you like to receive a summary of the results of this study? (Q58)

( ) Yes

( ) No

# Contact information (only if ‘draw’ or ‘summary’ = Yes)

If “Draw” = “Yes” then: You will be entered into the draw to win a $100 Amazon giftcard. (Q59)

If “Results” = “yes” then: We will email you a summary of the results when they are available. (Q60)

To facilitate this, please provide your email address below. This information will be kept separate from your survey responses and will not be reported or disclosed to any third parties. Your email address will not be included in the study database, it will only be kept for the purpose of contacting you (summary of results and/or draw), after which it will be destroyed.

Name: [Text area, required, min length 3, max length 75]

Email address: [Text area, required, email address]
